# Autoimmune musicogenic epilepsy associated with anti‐glutamic acid decarboxylase antibodies and Stiff‐person syndrome

**DOI:** 10.1002/ccr3.2538

**Published:** 2019-12-12

**Authors:** Joana Jesus‐Ribeiro, Alireza Bozorgi, Modhi Alkhaldi, Mahmoud Shaqfeh, Guadalupe Fernandez‐Baca Vaca, Bashar Katirji

**Affiliations:** ^1^ Neurology Department Coimbra University Hospital Center Coimbra Portugal; ^2^ Neurology Department University Hospitals Cleveland Medical Center and Case Western Reserve University School of Medicine Cleveland OH USA

**Keywords:** anti‐glutamic acid decarboxylase antibodies, autoimmune epilepsy, musicogenic seizure, reflex seizure, Stiff‐person syndrome, temporal lobe epilepsy

## Abstract

Epilepsy should be suspected in patients with Stiff‐person syndrome and new onset paroxysmal episodes. Musicogenic epilepsy may be a manifestation of anti‐GAD‐Ab spectrum, supporting an autoimmune workup in these patients. Appropriate treatment is not well established, and immunotherapy should be considered in patients with only partial response to antiepileptic drugs.

## Graphical abstract

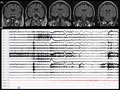

## 1 INTRODUCTION

Autoantibodies to glutamic acid decarboxylase (anti‐GAD‐Ab), the rate‐limiting enzyme for the synthesis of the inhibitory neurotransmitter gamma‐aminobutyric acid (GABA), are associated with a wide range of neurologic conditions depending on tissue distribution and epitope specificities.^1^ Besides the classical association with Stiff‐person syndrome (SPS), elevated anti‐GAD‐Ab was linked to unexplained adult‐onset focal epilepsy, mainly affecting the temporal lobe (TL) and frequently exhibiting drug‐resistant seizures.^1^, ^2^ Musicogenic reflexive seizures (MRS) were reported mainly in TL epilepsy, and its correlation to anti‐GAD‐Ab is unclear.^3^, ^4^ Indeed, the full clinical spectrum of anti‐GAD‐Ab, its specific seizure semiology, and appropriate treatment are not well established.

## 2 CASE REPORT

A 61‐year‐old right‐handed woman with seropositive SPS, diabetes mellitus (DM), epilepsy, and hypothyroidism.

Her SPS symptoms started in 2011 with frequent falls, truncal stiffness, and muscle spasms. She had an anti‐GAD‐Ab titer of 800 nmol/L (normal <0.02 nmol/L), with normal spine MRI, electromyogram, nerve conduction study, and muscle biopsy. She did well on symptomatic treatment (gabapentin and diazepam).

**Figure 1:**
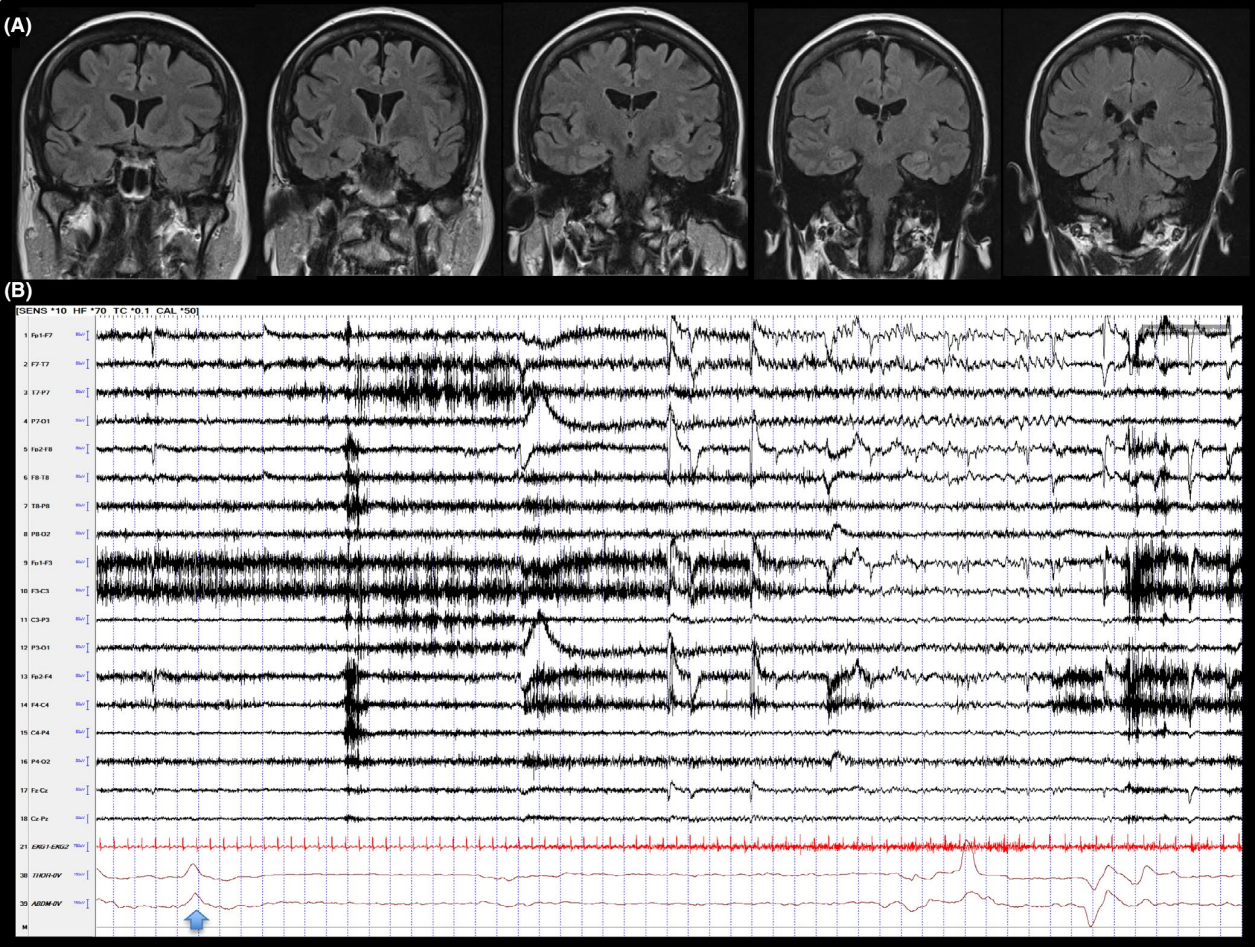
(A) Coronal Flair: 1.5T Brain MRI. (B) Ictal EEG: Musicogenic reflex seizure (apneic seizure followed by automotor seizure) arising from left mesial temporal lobe. Arrow: apnea onset, followed by theta rhythm over Sp1 (longitudinal bipolar montage). The time between music onset and seizure onset was 2 min and 26 sec

Her seizures started in 2014 when her husband witnessed episodes of unresponsiveness and automatisms. Most of her seizures would occur at a weekly basis while singing or listening to choral music at church, triggering a religious emotion. According to her husband, she would stop singing, stare and exhibit mouth and hand automatisms lasting for few minutes, followed by a period of confusion lasting for 2‐3 minutes. The patient had no recollection of the events. Occasionally, she would experience spontaneous seizures, which were not triggered by music. She was admitted to the Epilepsy Monitoring Unit, and she had four seizures arising from the left TL characterized by apnea followed by loss of awareness and automatisms (Figure 1.). All happened while listening to music or singing along. The epilepsy autoimmune panel in serum showed an elevated anti‐GAD‐Ab titer of 1280 nmol/L. Brain MRI was normal except for a minimal asymmetry of the temporal horns, right larger than left. She was treated with up‐titrating dose of levetiracetam. Her seizures were fairly controlled (one automotor seizure every two months) but she was not seizure free.

In 2018, she was diagnosed with insulin‐dependent DM. She was then started on intravenous immunoglobulin pulses, with improvement of the symptoms related to SPS, glycemic values, and seizure frequency.

## 3 DISCUSSION

We describe an association between MRS and SPS related to anti‐GAD‐Ab, which has not been previously reported. This case shows MRS as a distinctive epilepsy type that may be found in patients with SPS and anti‐GAD‐Ab, helping in the early identification of these patients.

Musicogenic epilepsy is a rare form of reflex epilepsy in which seizures are triggered by musical stimuli, ranging from simple tones to complex symphonic music.^4^, ^5^ A literature review between 1884 and 2018 found 123 cases of MRS. Only two cases were related to anti‐GAD‐Ab but none of them had SPS.^3^ Similar to our patient, these two patients had an adult‐onset TL epilepsy, with automotor seizures induced by different musicogenic triggers, as well as spontaneous seizures (Table 1). In general, MRS was mostly documented in patients with a temporal epileptogenic zone and described originating from dominant and nondominant hemispheres.^4^ None of the patients had musical training which was suggested to predispose to musicogenic epilepsy.^4^ The pathophysiology involved in MRS has not been well defined.^4^, ^5^ Nevertheless, most reports emphasized the emotional component as the causal factor in stimulation of the epileptogenic zone, implying a complex evoked response involving multiple cortical areas and association cortex rather than a pure auditory evoked response.^4^, ^5^

**Table 1:** Overview of case reports on anti‐GAD‐Ab and musicogenic reflexive seizures

Authors	N	Demographic data	Seizure semiology	Seizure frequency	Musicogenic triggers	Unprovoked seizures	Video‐EEG	Brain MRI	Other autoimmune manifestations	Anti‐GAD‐Ab titter (serum)	Other Antibodies	Antiepileptic drugs	Seizure frequency on antiepileptic drugs	Immunotherapy
Present case	1	61y, Female, right‐handed Onset: 58y	Apnea, automotor seizure	Weekly	Listening and singing religious music Emotional component	Yes	Left TL	Normal	Stiff‐person syndrome Hypothyroidism DM	1280 nmol/L	None	Levetiracetam	Every 2 mo	IVIG
Falip et al^3^	2	69y, Female, right‐handed Onset: 39y	Nocturnal GTCS Epigastric aura, automotor seizure and ictal speech	‐	Listening flamenco Emotional component	Yes	Right TL	Normal	DM	654.000 KUI/L^a^	None	Phenytoin Valproate Topiramate Phenobarbital Zonisamide	Monthly	None
		39y, Male, right‐handed Onset: 30y	Epigastric, psychic and olfactory aura Automotor seizure sGTCS	Daily	Playing rock music Emotional component	Yes	Left TL	Normal	DM Hypothyroidism	1117 KIU/L^a^	Antithyroid	Oxcarbazepine	1‐2/mo	IVIG

Abbreviations: anti‐GAD‐Ab, anti‐glutamic acid decarboxylase antibody; DM, diabetes mellitus; GTCS, generalized tonic‐clonic seizure; IVIG, Intravenous immunoglobulin; sGTCS, secondary generalized tonic‐clonic seizure; TL, temporal lobe; Y, years old.

a Positivity >5 KIU/L.

Two epileptic clinical scenarios related to anti‐GAD‐Ab have been described: (a) an acute epilepsy associated with limbic encephalitis; (b) a chronic slowly evolving focal epilepsy, that can become pharmacoresistant.^1^, ^6^ Anti‐GAD‐Ab titers did not correlate with the severity of the epilepsy.^1^ Our patient and the two similar patients published earlier would fit better under the second clinical scenario. MRI findings described in these cases were dependent upon the time course of the disease, with the first MRI being normal in the majority of the patients, but during the follow‐up mesial temporal lobe sclerosis were observed in some.^6^

All three responded partially to AEDs. Two patients were treated with immunotherapy, one with no improvement.^3^ Immunotherapy has been used in patients with SPS as well as epilepsy and anti‐GAD‐Ab with varying success.^1^, ^7^ Among 64 patients with focal epilepsy and anti‐GAD‐Ab, 42 (65.6%) received immunotherapy and 24 of these (57.1%) successfully responded to it (seizure reduction ≥50% or seizure freedom).

Epileptic seizures should be suspected in patients with SPS and new onset paroxysmal episodes. Autoimmune workup with anti‐GAD‐Ab determination should be performed in patients with MRS. Immunotherapy should be considered in epileptic patients with anti‐GAD‐Ab when they do not fully respond to AEDs.

## CONFLICT OF INTEREST

The authors have no conflicts of interest to declare.

## AUTHOR CONTRIBUTIONS

JJ‐R: involved in conception and design, analysis and interpretation of data, final approval of the version to be published, and agreed to be accountable for all aspects of the work. AB, MA, and MS: involved in acquisition of data, analysis and interpretation of data, drafting the manuscript, final approval of the version to be published and agreed to be accountable for all aspects of the work. GF‐BV and BK: involved in conception and design, revising the manuscript critically for important intellectual content, final approval of the version to be published, and agreed to be accountable for all aspects of the work.

## ETHICAL APPROVAL

Subject has given her written informed consent to publish this case.
